# Immunomodulatory Effects and Induction of Apoptosis by Different Molecular Weight Chitosan Oligosaccharides in Head Kidney Macrophages From Blunt Snout Bream (*Megalobrama amblycephala*)

**DOI:** 10.3389/fimmu.2019.00869

**Published:** 2019-05-15

**Authors:** Changsong Wu, Yishan Dai, Gailing Yuan, Jianguo Su, Xiaoling Liu

**Affiliations:** ^1^Department of Aquatic Animal Medicine, College of Fisheries, Huazhong Agricultural University, Wuhan, China; ^2^Hubei Provincial Engineering Laboratory for Pond Aquaculture, Hubei Engineering Technology Research Center for Aquatic Animal Disease Control and Prevention, Wuhan, China

**Keywords:** *Megalobrama amblycephala*, Chitosan oligosaccharide, macrophage, transcriptome, apoptosis, P53 pathway

## Abstract

Prophylactic administration of immunopotentiators has been tested and practiced as one of the most promising disease prevention methods in aquaculture. Chitosan oligosaccharide (COS), as an ideal immunopotentiator, is mainly used as feed additives in aquaculture, and the antimicrobial and immune enhancement effects are highly correlated with molecular weight (MW), but little is known about the mechanisms in teleost. Here, we isolated and purified macrophages in head kidney from blunt snout bream (*Megalobrama amblycephala*), stimulated them with three different MW (~500 Da, ~1000 Da and 2000~3000 Da) COSs, performed RNA-sequencing, global transcriptional analyses, and verification by quantitative real-time PCR (qRT-PCR) and immunofluorescent staining methods. Differential expression gene (DEG) analysis indicated that gene expression patterns are different and the proportion of unique genes are relatively high in different treatment groups. Biological process and gene set enrichment analysis (GSEA) demonstrated that all three COSs activate resting macrophages, but the degrees are different. Weighted gene co-expression network analysis (WGCNA) reflected gene modules correlated to MW, the module hub genes and top GO terms showed the activation of macrophage was positively correlated with the MW, and larger MW COS activated cell death associated GO terms. Further use of the screening and enrichment functions of STRING and Pfam databases discovered that apoptosis-related pathways and protein families were activated, such as the P53 pathway and caspase protein family. qRT-PCR results showed that as the stimulation time extends, the innate immune-related and P53 pathways are gradually activated, and the degree of activation is positively correlated with the stimulation time. In addition, apoptosis was detected by immunofluorescent staining in three groups. Therefore, the use of COS has two sides—it can activate the immune system against pathogen invasion, but with the increase in stimulation time and MW, macrophage apoptosis is induced, which may be caused by abnormal replication of DNA and excessive inflammation. This study provides a theoretical basis for the rational use of COS as an immunopotentiator in aquaculture.

## Introduction

In recent years, with the increasing scale and density of aquaculture industry, infections by a variety of fish pathogens have become more frequent. The negative effects of chemotherapeutants have become increasingly prominent, such as increased resistance of pathogens, decreased cellular, and humoral immune functions of aquatic animals, and drug residues directly threatening human health and safety. Therefore, the prevention and control of aquatic animals from the perspective of immunology have gradually become a research hotspot. Immunopotentiators are a new type of fishery drug that activate the body's immune function and enhance its resistance to infectious diseases (1). Their main immunomodulating mechanism is to act on the cell surface receptors and enable cells to produce cytokines to clear pathogens (2). The application of immunopotentiators is important for controlling fish diseases in aquaculture (3). Chitosan is an important immunopotentiator and is the only alkaline polysaccharide in nature. It has been reported to regulate the function of isolating immune cells *in vitro* (4).

COS, a polymer composed of deacetylated glucosamine units and β-(1-4)-linked N-acetyl-D-glucosamine (GlcNAc), has a polymerization degree (DP) of 2~20 and an average MW <3,900 Da (5, 6). Due to its water solubility, non-toxicity, superior biocompatibility and adsorption properties, the potential application of COS as dietary supplements or medications has received considerable interest (7). In recent years, more and more research has been devoted to exploring the biological significance of COS application. The results showed that COSs have exhibited versatile biological functions, including anti-oxidative, anti-inflammation anti-microbial, anti-tumor, and anti-coagulant properties (8–10).

Macrophages are critical immune cells that play pivotal roles in both defense and immune homeostasis. Studies have shown that COS is recognized by macrophages and regulates macrophage function as one of the important ways to play an immunomodulatory role (11–13). On the one hand, COS activates resting macrophages to release NO and cytokines. NO has a cytotoxic effect, and a large amount of NO released can kill microorganisms, parasites, and tumor cells (14). Cytokines play an important role in inflammatory response and immune response and regulate both innate and adaptive immunity. It has been reported that COS stimulates resting macrophages to promote the secretion of Th1 cytokines (15). On the other hand, COS can also weaken activated macrophages and inhibit inflammation-related gene secretion (16). In addition, some studies have shown that the biological functions of COS are closely related to MW. COS with DPs of 6–8 (or 4–7 ~ 5–7) have good antibacterial activity, immunopotentiating effect, and antitumor activity. About 1,700 Da MW COS is suitable for patients with hyperlipidemia; this amount can reduce blood sugar and improve antioxidant ability (17–19). In the study of mammals, the immunoregulatory mechanisms of COS involve the modulation of several important pathways including the suppression of nuclear factor kappa B (NF-κB) and mitogen-activated protein kinases (MAPK), and the activation of AMP-activated protein kinase (AMPK) (20–23). However, the studies of mechanisms are still in the initial stage in bony fish.

In this study, we used three different MW COSs to stimulate the head kidney macrophages of blunt snout bream *in vitro*. Then, the similarities and differences of biological functions of COSs were compared by high-throughput sequencing and bioinformatics analysis. We conducted experimental validation for some important findings. We expected to provide new ideas for the development and utilization of new immunopotentiator in aquaculture.

## Materials and Methods

### Fish Sampling

Blunt snout bream (*M. amblycephala*), ranging from 400 g to 500 g in weight, were obtained from a fish farm located in Hubei Province, China and kept in a recirculating freshwater system at 25–26°C with a natural photoperiod. The animals were fed twice per day with a commercial pellet diet (Haida, Hubei, China) amounting to 3% of body weight. The study was approved by the Institutional Animal Care and Use Ethics Committee of Huazhong Agricultural University.

### Isolation of Head-Kidney Macrophages

Blunt snout bream head kidney macrophages were isolated as described previously with slight modifications (24). Briefly, fish were anesthetized with MS222 (Syndel Laboratories, Ltd., Canada) and the head kidney was removed aseptically and passed through a 100 lm mesh (Falcon, Becton Dickinson) in Leibovitz medium (L-15) (Invitrogen, USA) containing 2% fetal bovine serum (FBS) (Gibco, USA) and 200 IU/ml penicillin plus streptomycin (Amresco, USA). The resulting cell suspension was layered onto a 34%/51% Percoll (Pharmacia, Uppsala, Sweden) density gradient and centrifuged at 400 g for 30 min at 4°C. The interface was collected and the cells were washed twice with L-15 at 400 g for 10 min at 4°C before being resuspended to 1 × 10^7^ cells/ml in L-15 containing 10% FBS.

### Macrophages Stimulated With COSs and GlcNAc

A total of 2 ml of the macrophage suspension (1 × 10^7^ cells/ml in L-15 containing 10% FBS) was dispensed into each well of a 6-well plate. After 12 h incubation at 28°C, the non-adherent cells were washed off. COS with MWs ~500 Da (COS3), ~1000 Da (COS6), 2000~3000 Da (COS13-19), and GlcNAc (N-acetyl-D-glucosamine, the monomeric unit of the polymer chitosan) were added to each well (20 μg/ml), respectively (4). PBS was added to each well as the control group. Then, these samples were further incubated for 4, 8, and 16 h at 28°C.

### RNA Extraction and cDNA Synthesis

The total RNA from each well was collected with a High Pure RNA Isolation Kit (Roche, Basel, Switzerland) following the manufacturer's instructions. Quality and quantity of the extracted RNA were assessed by electrophoresis in 1% agarose gels and with NanoDrop 2000 spectrometer (Thermo Scientific, USA), using the A260/A280 > 1.8 criterion as the acceptable quality threshold. Approximately 1 μg of total RNA was used to synthesize the first strand cDNA using the PrimeScript® RT reagent Kit with gDNA Eraser (TaKaRa, China) according to the manufacturer's protocols and then stored at −20°C.

### cDNA Library Preparation, Illumina Sequencing, and Data Analysis After Stimulation for 4 h

Poly(A)^+^ RNA was purified from total cellular RNA using poly(dT) oligo-attached magnetic beads, and full-length cDNAs were synthesized with a KAPA Stranded RNA-Seq Library Preparation Kit (Illumina Inc., USA) according to the manufacturer's protocol. The cDNA libraries were sequenced on the Illumina Xten genomic sequencing platform to generate 150-bp paired-end reads, by Wuhan Whbioacme Co. Ltd. Raw reads were first filtered to remove the adaptor and bases of low quality by Trimmomatic (25). Filtered reads were aligned to the *M. amblycephala* genome by HISAT2 (26). The sequencing quality of the raw data and mapped reads ratio after quality control for 15 samples are shown in Dataset S1. Although the whole genomic sequence of *M. amblycephala* has been published, the resulting document of gene structure prediction has not been made public (27). Therefore, Geta software was used to make gene structure prediction, and we re-obtained the genomic structure information (Dataset S2) and gene sequence (Dataset S3) (https://github.com/chenlianfu/geta). Subsequently, the newly obtained protein sequence (Dataset S4) was compared with the published sequence using Busco (28). The proportions of homologous genes in the newly predicted and published sequences were 89.7 and 88.2%, respectively. The result show that our prediction is more accurate. Gene expression was quantified with cufflinks, expression values were normalized for library size and differentially expressed genes were considered with DESeq2 (29, 30). All raw data of the results of this article are available in the NCBI Sequence Read Archive Database (http://www.ncbi.nlm.nih.gov/Traces/sra/) under accession number SRP169988.

### GO Term and KEGG Pathway Enrichment Analyses

To analyze the potential functions of genes, we first re-annotated the genes of *M. amblycephala*. Briefly, blunt snout bream genes were mapped to multiple public databases such as NCBI non-redundant (NR), Gene Ontology (GO), Swiss-Prot/UniProt, and the Kyoto Encyclopedia of Genes and Genomes (KEGG) databases. Using all the genes as background, we used the numbers of DEGs to calculate the *P*-value (<0.05), which represent the significance of enriched GO terms/KEGG pathways and control the false discovery rate, respectively. The *p*-values were calculated by Fisher's exact test.

### Gene Set Enrichment Analysis (GSEA)

To investigate the stimulation effect of COS on various biological function gene sets in macrophages, differences in gene mRNA expression levels of biological functional annotation and pathways between control and COS stimulation groups with different MW were analyzed by GSEA (http://software.broadinstitute.org/gsea/downloads.jsp). For use with GSEA software, the database file (Dataset S5) about KEGG pathway enrichment analyses of *M. amblycephala* was built*. P*-value < 0.05 was chosen as the cut-off criteria.

### Weighted Gene Co-expression Network Analysis and Protein-Protein Interaction Network Analysis

The weighted correlation network was constructed using the freely accessible R software package as previously described (31, 32). We selected the 10,000 most variant genes for WGCNA analysis. Three different ways can be selected to construct the network and identify modules according to different needs. In our study, the one-step function was used for network construction and detection of consensus modules. The modules were filtered using the following criteria: Pearson *P* > 0.8 and *P*-value < 0.001. Furthermore, we extracted a subnetwork with module genes from the high quality STRING protein interaction database (combined score ≥ 600) (33). Since the STRING database weights and integrates information from numerous sources, including experimental repositories, computational prediction methods, and public text collections, we only parsed the high-quality part of it, hoping to get a convincing interaction subnetwork of our module genes. The subnetwork was illustrated with Gephi (https://gephi.org).

### Quantitative Real-Time PCR

Quantitative real-time PCR (qRT-PCR) was used to investigate the target gene expression patterns in different groups and different time points (0 h, 4 h, 8 h, and 16 h) of macrophages after COS stimulation. Primers used for qRT-PCR of this experiment are given in Table 1. The qRT-PCR mixture reaction volume was 20 μl, containing 10 μl LightCycler® 480 SYBR Green I Master, 7.4 μl ddH_2_O, 0.8 μl of each primer (10 mM), and 1 μl cDNA template. The reactions were performed using LightCycler®480 II (Roche Diagnostics GmbH, USA) according to the procedure as follows: preincubation at 95°C for 5 min, then 40 cycles at 95°C for 5 s, 55°C for 20 s, and 72°C for 20 s. Each sample was tested in triplicate. Specificity of the amplified target gene was assessed using dissociation curve analysis. The target gene relative expression levels vs. the β-actin gene (was selected as the reference gene) was calculated according to the 2^−ΔΔCT^ method. To determine the relative fold change of the target gene at different time points, the expression value was normalized using the corresponding control group.

**Table 1:** Primers used for qRT-PCR in this study.

**Gene name**	**Forward primer (5**′**-3**′**)**	**Reverse primer (5**′**-3**′**)**	**Gene number**
ERK1	TCCTGCGAGGGCTGAAATAC	TCCGGTGTGGTCATGTTCTG	MK315044
ERK2	CCCTAAAGCGTTGGACCTGT	AGGTTAAACGGAGCCTCAGC	MK315045
Jnk1	AGCACCCCTACATCAACGTG	CGTTTTTCGTTCGCTCCTCC	MK315047
P38α	TGGGAGCGGATCTCAACAAC	TCAGGCCAGCTGAATGGATG	MK315052
P38β	TGTCGACAAGACCGGGATTC	CTGCTGTGGATGAGGGACTG	MK315053
Fos	GCTGCAAGCTGAAACTGACC	CGATAGGTGAGACGGATGGC	MK315046
Junb	ACTTGAACCTGACGGAACCC	CTTCCTGCTCGTCGGTGATT	MK315048
Jund	AGGAAGAAGCTGCGTAACCG	TTGCTCTCTCAGAACGCTCG	MK315049
NF-κB1	TGGATGGAGGGGCAGATGTA	AAGTGCGCTCAGTTTGCTTG	MK315050
NF-κB2	AACTACCAGTTGAGCGGTGG	GGTCACTGCAGGATTTCCCA	MK315051
TNF-α	CCGCTGCTGTCTGCTTCA	GCCTGGTCCTGGTTCACTCT	HQ696609.1
ATR	GGAGACGGCCAACTTTGAGA	CGAATGAGGTTGCCCTGTCT	MK315042
ATM	CCCGGAGAACCGAATGTTGA	AATCCCTCCAAACGAGCCAG	MK315041
Bax	CCGGCTTGTCATCAAGGCTA	GTGGGGGTGCCAAAATAGGA	MK315043
P53	TGCTGACTGAACAGCCTCAG	GAACTGGACACGTTTTCGCC	MK315040
Caspase3	GGTGGATGCTATGCCTCAGT	CCATTGCGTTGGTTCATGCC	KY006115.1
β-actin	GTGCCAGGTGCCAAGTAGC	AAGCCCAAGATATGCAGGAGT	ADV57164.1

### Analysis of Apoptosis by Annexin V-FITC

Annexin V-FITC/PI double staining was used to evaluate cell apoptosis after stimulation for 16 h, strictly following the procedures of the Annexin V-FITC Apoptosis Detection Kit (Beyotime Institute of Biotechnology, China). Briefly, macrophages were seeded in 24-well plates and cultured as before, then treated with COSs (20 μg/ml) for 16 h. After treatments, the cell culture medium was removed, and it was washed once with PBS. We then added 195 μl media binding reagent, 5 μl Annexin V-FITC and 10 μl PI to stain the cells. Finally, cells were incubated at 28°C for 20 min in the dark.

### Statistical Analysis

In the present study, data generated by qRT-PCR were presented as the means of three biological replicates ± SE. The statistical significance was assessed by two-tailed independent *t*-test. *P* < 0.05 value was considered to be statistically significant difference and *P* < 0.01 value as extreme difference.

## Result

### Expression of Differentially Expressed Genes (DEGs) After COS Stimulation of Head Kidney Macrophages

**Figure 1:**
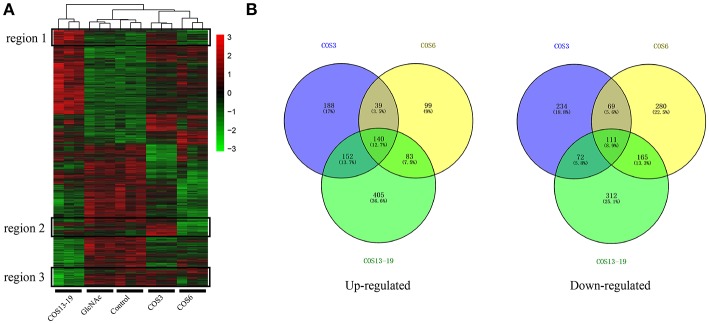
Differentially expressed gene (DEG) analysis of different MW COS-stimulated macrophages (*P* < 0.001). **(A)** A heatmap was used to classify the DEG expression patterns in all groups, the x-axis represents the experimental conditions (4 h). **(B)** Venn diagrams of DEGs in COS3, COS6, and COS13-19 groups. Left panel: up-regulated genes; right panel: down-regulated genes.

We analyzed the DEGs among the control, GlcNAc, COS3, COS6, and COS13-19 groups with DESeq2. Compared with the control group, the COS3, COS6, and COS13-19 groups contained 1005, 986, and 1440 DEGs, respectively (*P*-value < 0.001). However, there were only 10 DEGs between the control and GlcNAc group, and we think that GlcNAc could not activate resting macrophages (Figure S1). These results were clearly visualized by clustering the samples by differential treatment and by constructing a Venn diagram of the DEGs (Figure 1). Some DEGs in certain stimulated group were upregulated but in others they were downregulated or showed no difference, like the regions genes in Figure 1A. Interestingly, different stimulated groups had different proportions of unique genes in up- or downregulated genes, especially in the COS13-19 group (Figure 1B). This may suggest that COSs with different MWs have different effects on macrophages from blunt snout bream head kidney.

### Functional Enrichment Analysis of DEGs

**Figure 2:**
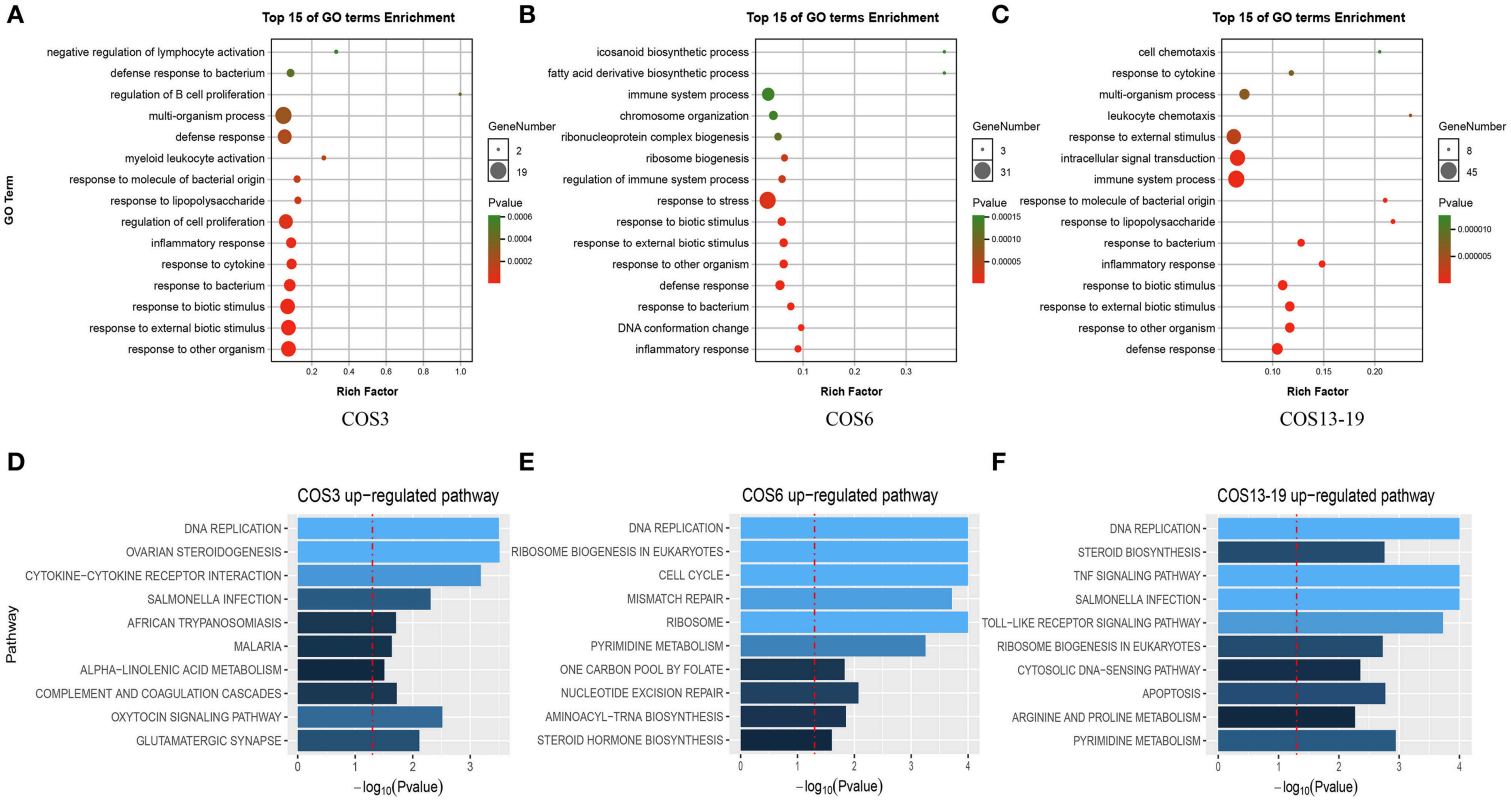
Functional enrichment analysis identified major biological processes and pathways in COS3, COS6, and COS13-19 groups. **(A–C)** GO enrichment analysis of upregulated genes in COS3, COS6, and COS13-19, respectively. **(D–F)** Gene set enrichment analysis (GSEA) of all genes in COS3, COS6, and COS13-19.

There was a clear difference in the number of DEGs between the COS3, COS6, and COS13-19 groups compared with the control. Therefore, we performed Gene Ontology (GO) term and KEGG pathway analysis to filter the biological processes and pathways. The top 15 GO terms (in descending order of the *P*-value) of the three groups are shown in Figure 2. The common enrichment GO terms among the three stimulation groups are defense response, inflammatory response, response to bacterium, response to biotic stimulus, response to external biotic stimulus, and response to other organisms (Figures 2A–C). In addition, immune-related responses were enriched in one or two groups, mainly including immune system process (COS6 and COS13-19), regulation of immune system process (COS6), and leukocyte chemotaxis (COS13-19). In the GSEA analysis of KEGG enrichment, the top 10 KEGG pathways (in descending order of the NES) of the three groups are shown (Figures 2D–F). Some of the pathways associated with pathogenic infections were significantly enriched, including salmonella infection (COS3 and COS13-19), African trypanosomiasis (COS3), and malaria (COS3). Immune-related pathways were enriched in COS13-19 groups, such as TNF signaling pathway and Toll-like receptor signaling pathway. Notably, DNA replication was the most prominent pathway in the three groups (Figure S2). This may reflect a common characteristic of COS-stimulated macrophages.

### Weighted Gene Network Co-expression Analysis of COSs Stimulated Macrophages

Gene co-expression network analysis relies on the assumption that a strong correlation of mRNA expression levels for a group of genes suggests that these genes work cooperatively on features. A gene linkage in a network is simply the number of other genes that are expressed in relation to the gene. The network was initially constructed using the component of the weighted gene co-expression network (WGCNA) method (31, 32). This method has been widely used to construct a weighted gene co-expression network based on absolute Pearson correlation coefficient between gene expression and expression levels to detect gene clusters correlated with a trait (34, 35).

**Figure 3:**
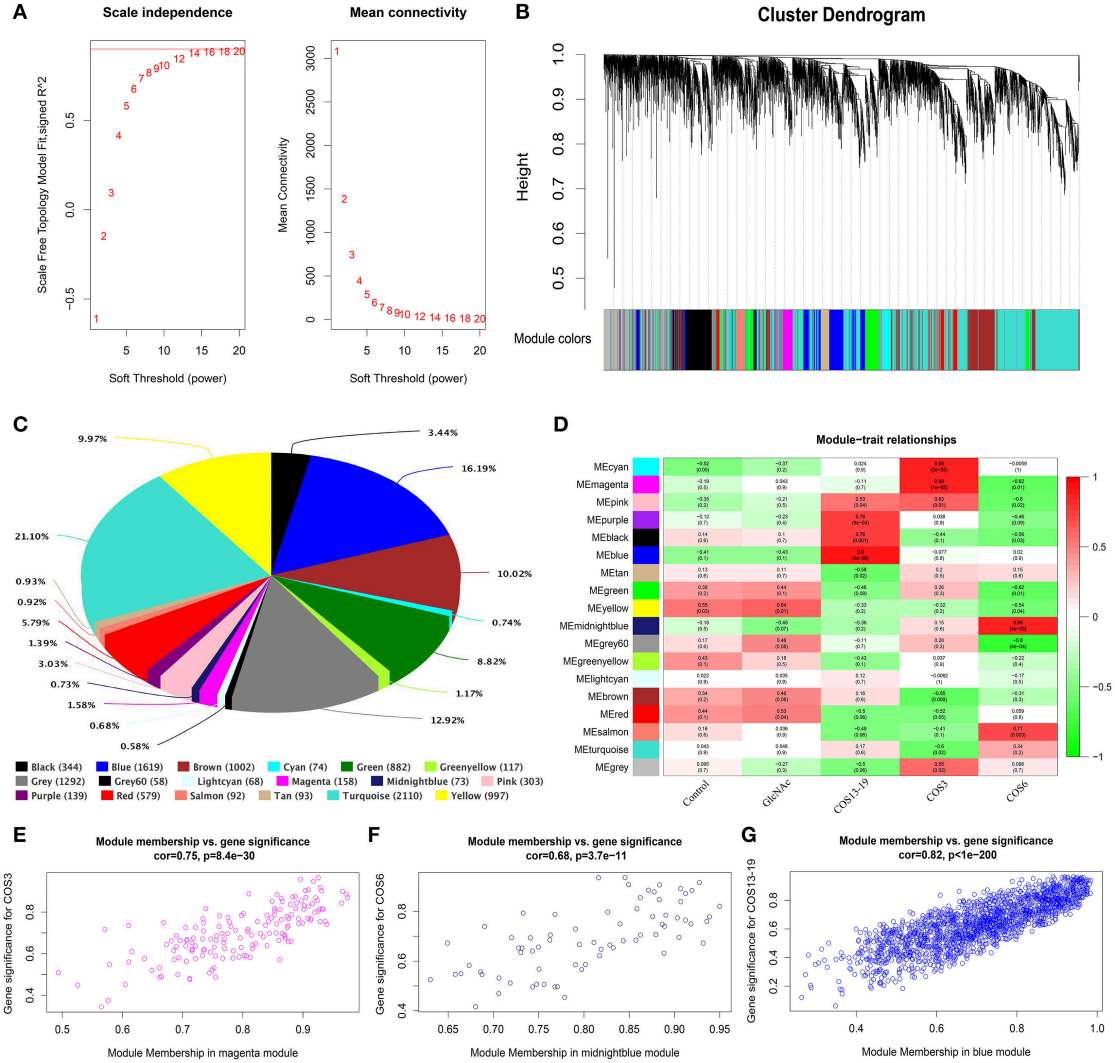
Weighted gene co-expression network analysis (WGCNA) yields behaviorally relevant modules. **(A)** Network topology for different soft-thresholding powers. Numbers in the plots indicate the corresponding soft thresholding powers. The approximate scale-free topology can be attained at the soft-thresholding power of 14. **(B)** Gene dendrogram obtained by clustering the dissimilarity based on consensus topological overlap, with the corresponding module colors indicated by the color row. Each colored row represents a color-coded module which contains a group of highly connected genes. In total, 17 modules were identified. **(C)** Pie chart visualizing the number and percentage of genes in each module. **(D)** Correlation between modules and traits. The upper number in each cell refers to the correlation coefficient of each module in the trait, and the lower number is the corresponding *P*-value. Red represents high adjacency (positive correlation) and green represents low adjacency (negative correlation). **(E–G)** Scatter plots of gene significance (GS) for COS3, COS6, and COS13-19 groups vs. the module membership (MM) in the magenta, midnightblue, and blue modules, respectively. Pearson's r (“cor”) and *P*-value as determined by Fisher's z-transformation are indicated above each plot.

In total, one control group and four treatment groups contained 15 samples, and 10,000 genes were used to construct the gene co-expression network. As 14 is the lowest value that allows obtaining more than 80% similarities in topology models of five groups (Figure 3A), a soft threshold of 14 was performed, resulting in the discovery of 17 significant modules (Figure 3B). The number and percentage of genes contained in different modules are shown in Figure 3C. Among these modules, the cyan and magenta modules were significantly correlated in the COS3 group, the midnightblue and grey60 modules were significantly correlated in the COS6 group, and the blue module was significantly correlated in the COS13-19 group (Pearson *P* > 0.8 and *P*-value < 0.001, Figure 3D). Additionally, gene intramodular analysis of GS and MM in the 5 modules followed. Because GS and MM exhibit significant correlation, the present finding implies that the genes in the module tend to be highly correlated with COS stimulated macrophages, and the magenta, midnightblue and blue modules were the most relevant modules in the COS3, COS6, and COS13-19 groups, respectively (Figures 3E-G). This may indicate that these genes contributing to macrophages status in the three groups, so we selected the three modules for further analysis.

### Network Construction and Analysis of Selected Modules

**Figure 4:**
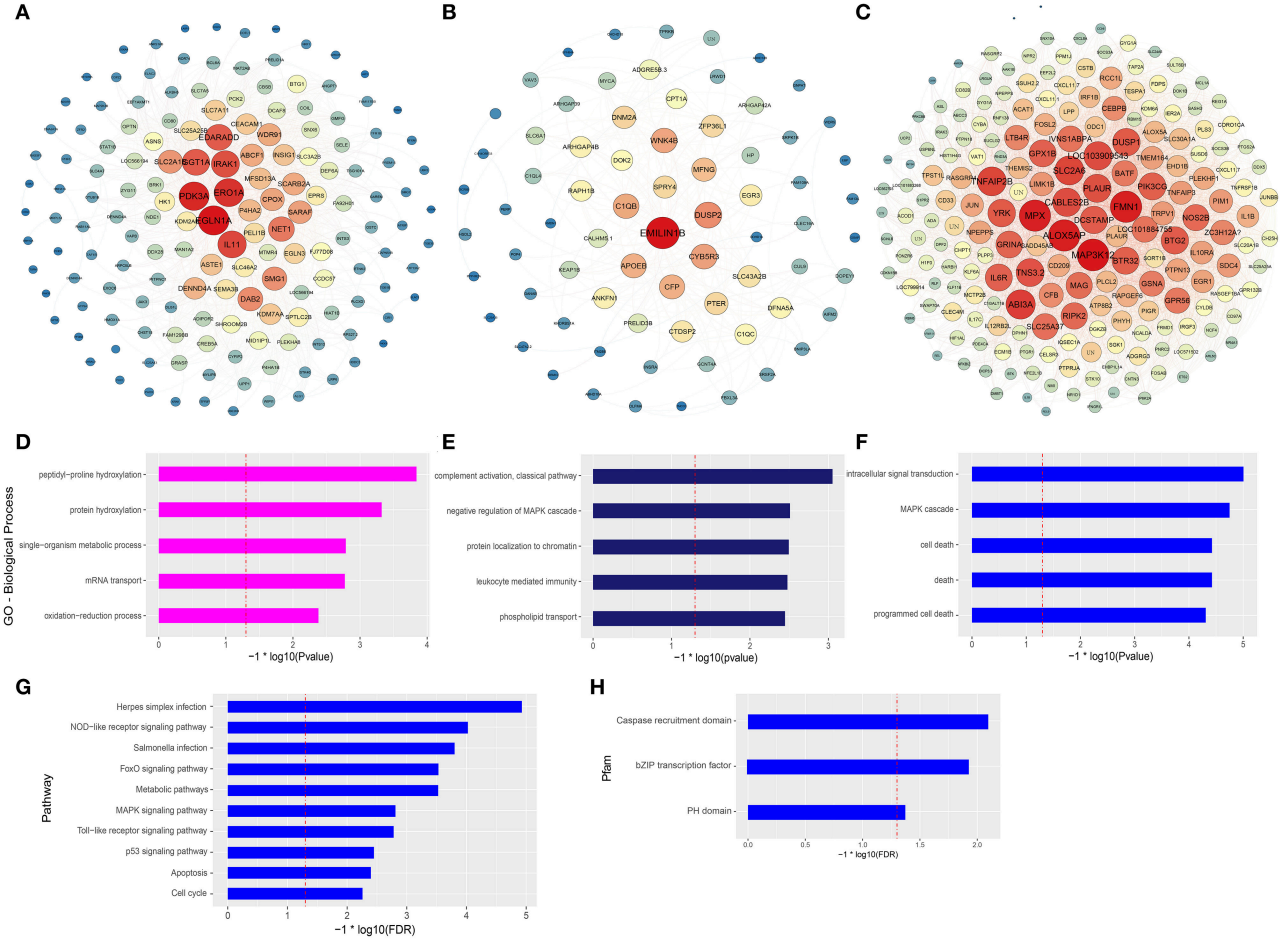
Gene network and enrichment analysis of selected modules. **(A–C)** Top hub genes of the magenta, midnightblue and blue modules are shown. Gene importance was assigned according to circle diameter, and color depth. **(D–F)** GO enrichment analysis of magenta, midnightblue, and blue modules genes. Top 5 GO biological process terms are shown. **(G)** KEGG pathway and **(H)** protein families and domain enrichment analysis of blue module genes screened from the STRING and Pfam databases.

The co-expression networks of top ranked genes for the magenta, midnightblue, and blue modules were constructed as shown in Figures 4A–C. The 200 strongest connection genes within the blue module were selected to show their connections and confirm hub genes. Within each network, color depth, font sizes, and node sizes are proportional to their connectivity (sum of in-module degrees). To study the biological functions of the magenta, midnightblue and blue modules, we implemented GO term enrichment analysis. For the magenta module, the top 5 enriched GO terms are shown in Figure 4D, including peptidyl-proline hydroxylation, protein hydroxylation, single-organism metabolic process, mRNA transport, and oxidationreduction process. Additionally, in Figure 4A, the hub genes like EGLN1A, ERO1A, EGLN3, P4HA2, and CPOX were proven to be related to intracellular oxygen concentration. The post-translational formation of 4-hydroxyproline in hypoxia-inducible factor (HIF) alpha proteins is catalyzed by EGLN1A (36). For the midnightblue module, the top 5 GO terms—including the classical pathway of complement activation, negative regulation of MAPK cascade, protein localization to chromatin, leukocyte mediated immunity, and phospholipid transport—were enriched (Figure 4E). The hub genes in this module are related to the innate immune system and cell proliferation, such as DUSP2, C1QB, CFP, SPRY4, C1QC, EMILIN1B, WNK4B, ZFP36L1, EGR3, and so on (Figure 4B). In the blue module, intracellular signal transduction, MAPK cascade, cell death, death, and programmed cell death GO terms were enriched (Figure 4F), as were the hub genes associated with innate immunity and antitumor activity, such as MAP3K12, FMN1, MPX, ABI3A, SLC2A6, TNFAIP2B, DUSP1, NOS2B, JUN, IL1β, and so on (Figure 4C). It is worth noting that three GO terms associated with death were significantly enriched in the blue module. In order to further explore the potential function of macrophages stimulated by COS13-19, the biological characteristics of the blue module were examined using existing data on protein-protein interactions, which have been gathered in the publicly available STRING database. We selected the proteins with an interaction score ≥ 0.4 in PPI network to perform KEGG pathway enrichment analysis and protein family and domain accurate classification. The top 10 pathways were shown in Figure 4G; these pathways were associated with infectious diseases, the immune system, cell growth and death, signal transduction, and metabolism. Interestingly, the enriched results of protein families and domains included caspase recruitment domain, bZIP transcription factor, and PH domain (Figure 4H). These results may imply that COS13-19 not only activates the immune system of macrophages, but also regulates cell proliferation and apoptosis through some pathways, such as the FoxO, MAPK, and p53 signaling pathways and cell cycle. Overall, the identification of some genes within the three modules that are known to regulate immune system, cell growth and death, and the gene expression network analysis using the WGCNA approach provides valuable insight into the pathways regulating macrophages which contribute to the COSs stimulated macrophages.

### COSs Activated MAPK and NF-κB Signaling Pathways in Blunt Snout Bream Head Kidney Macrophages

**Figure 5:**
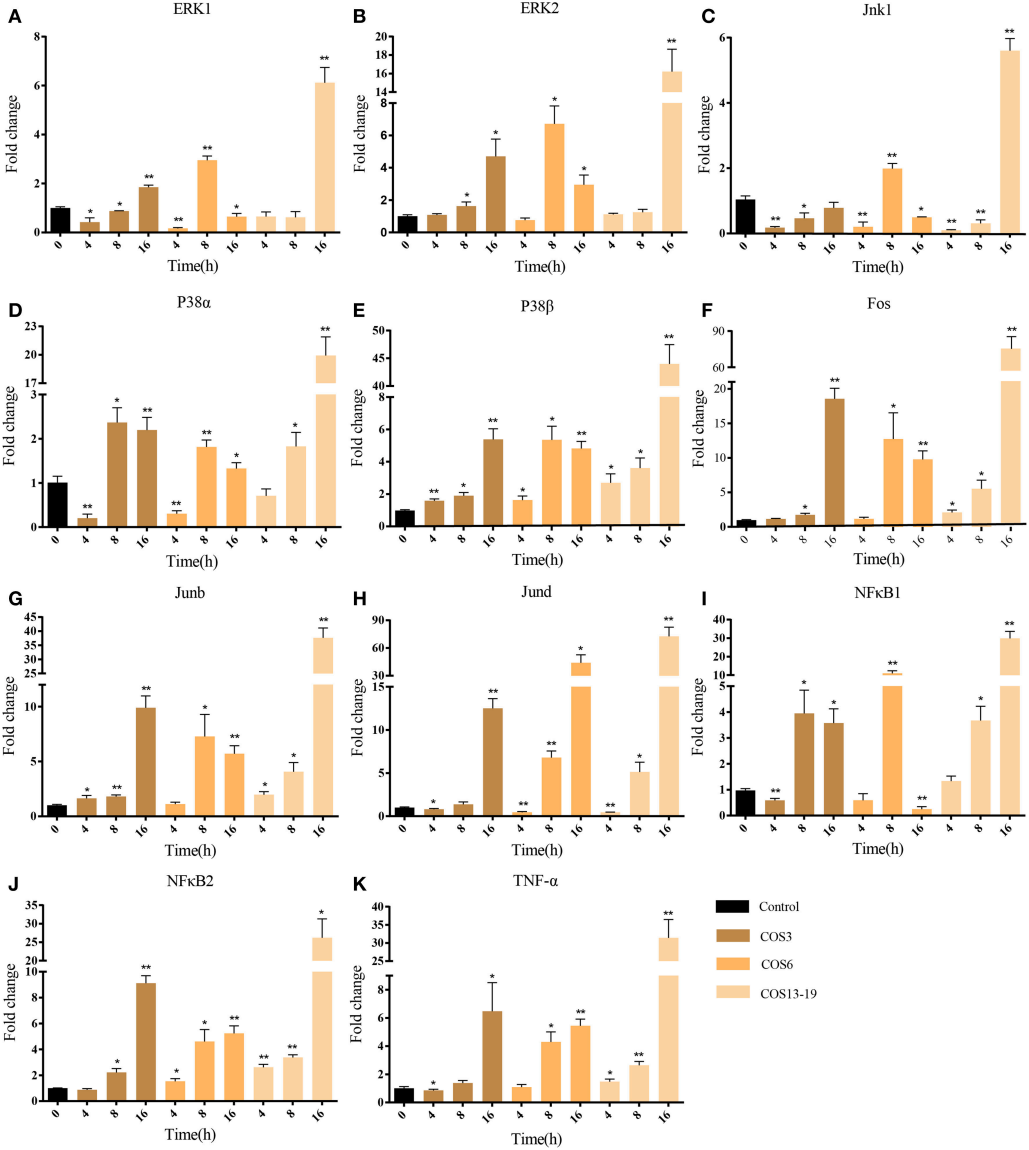
qRT-PCR identified the key gene expressions of the MAPK and NF-κB pathways. **(A–H)** The key genes of the MAPK pathway. **(I–K)** The key genes of NF-κB pathway. The samples were analyzed at 0, 4, 8, and 16 h post-stimulation. β-actin was used as internal reference. Each experiment was executed in triplicate. Data are shown as mean ± SE (*N* = 3). The asterisk indicates significant difference (**P < 0.01, *P < 0.05) compared with 0 h (set as 1).

In mammalian studies, COS can promote the secretion of cytokines by resting macrophages and play an important role in inflammatory and immune response. The two most studied and identified pathways are the MAPK and NF-κB signaling pathways (4, 15, 37). In our results, the MAPK pathway and NF-κB upstream pathways were enriched. To further evaluate our results, we selected macrophages which qwew stimulated at different time points (4, 8, and 16 h) and key genes in the two pathways for qRT-PCR analysis. As shown in Figure 5, ERK1/2, Jnk1, P38α, P38β, Fos, Junb, and Jund are the key genes for MAPK pathway (Figures 5A-H). The qRT-PCR results showed that COS could activate the MAPK pathway through p38β at the early stage (4 h) of macrophage stimulation, and then upregulated the other genes. In the NF-κB pathway (Figures 5I-K), NF-κB2 was significantly upregulated in the COS6 and COS13-19 groups at 4 h, and the expression of NF-κB1 and inflammatory cytokine TNF-α was upregulated. These results again demonstrate that MAPK and NF-κB signaling pathways are activated in COSs stimulated macrophages.

### COS Induced Apoptosis of Macrophages Via P53 Pathway

**Figure 6:**
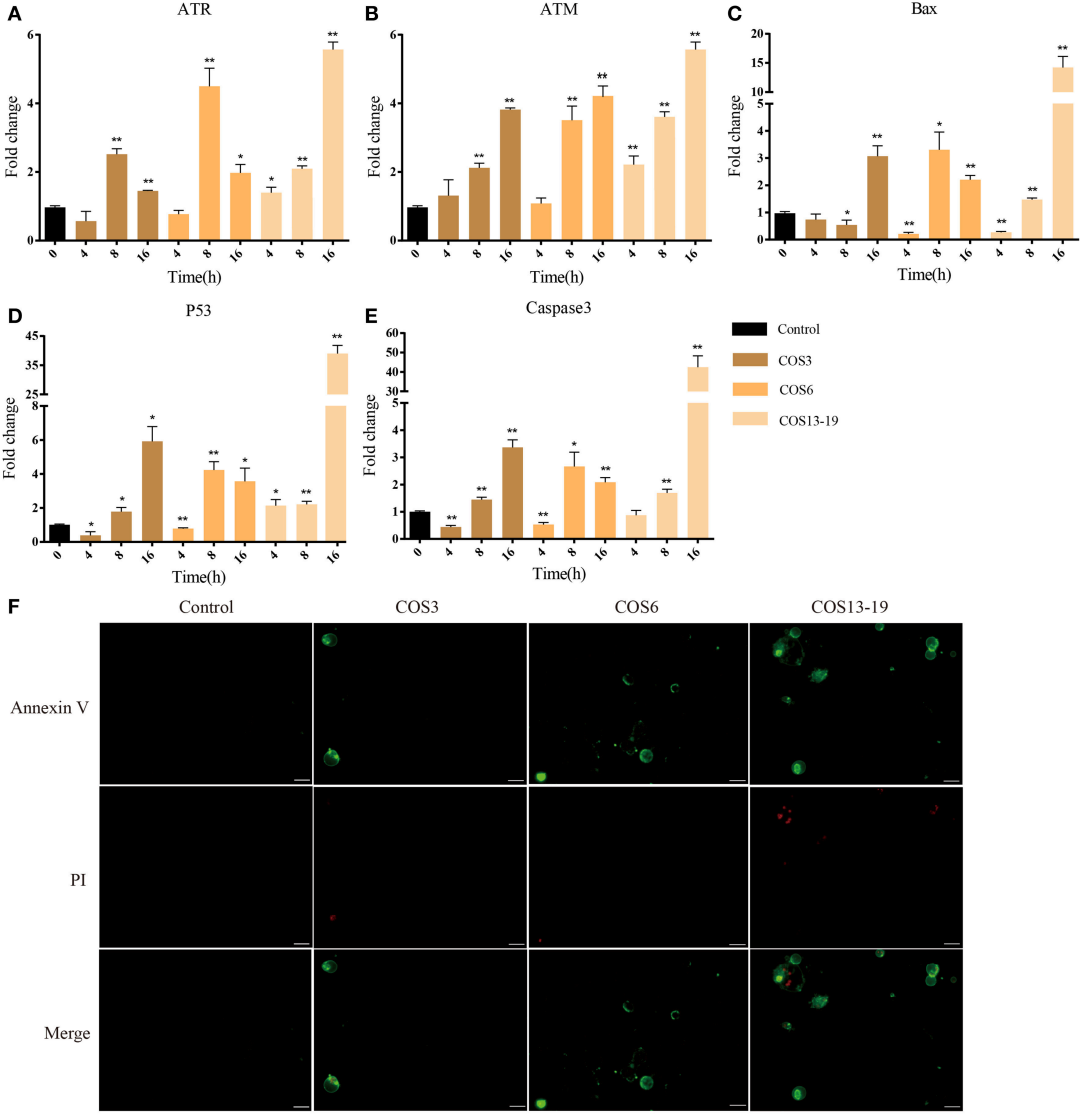
COSs induce apoptosis of blunt snout bream head kidney macrophages via the P53 pathway *in vitro*. **(A–E)** qRT-PCR identified the expression of key genes of the P53 pathway. The samples were analyzed at 0, 4, 8, and 16 h post-stimulation. β-actin was used as internal reference. Each experiment was executed in triplicate. Data were shown as mean ± SE (*N* = 3). The asterisk indicates significant difference (**P < 0.01, *P < 0.05) compared with 0 h (set as 1). **(F)** Cells were cultured for 16 h at 28°C after stimulation and then stained. Annexin V is the green fluorescent marker, labeling cells in early apoptosis. PI (Propidium Iodide) is the red fluorescent marker, labeling cells in late apoptosis. Bars: 25 μm.

P53 activation not only leads to cell cycle arrest, but also participates in cell apoptosis, which leads to two distinct results: the former provides the cells with the possibility of initiating repair and reversing the damage. The latter is lethal to cells. Therefore, p53 is considered to be one of the key factors determining cell survival and death (38). In our results, the P53 pathway, cell death-related GO terms, and caspase recruitment domain were enriched. To further verify our results, qRT-PCR and immunofluorescent staining methods were used. These results are shown in Figure 6. In the early stages of stimulation (4h), only the ATR and P53 genes were significantly upregulated, and Bax and Caspase 3 were subsequently significantly upregulated (Figures 6A–E). The results of immunofluorescence staining showed apoptosis occurred after 16 h of stimulation, especially in the COS13-19 group (Figure 6F and Figure S3). These results implied that COS could promotes macrophage apoptosis through the p53 signaling pathway.

## Discussion

Chitosan oligosaccharides (COSs) are natural oligomers derived from chitosan and are the most abundant carbohydrate polymers after cellulose. The biological activities of COSs are dependent on its structural characteristics such as the DP and MW. The higher oligosaccharides (pentamer or larger oligomers) possess various physiological activities such as antimicrobial, antifungal, anti-tumor, radical scavenging, and immuno-stimulating activity (4, 39). In mammalian studies, the immune effects of COS have been studied intensively at the individual and cellular levels. However, the studies in bony fish were mainly focused on feed additives and adjuvants, and the mechanism of action is not clear. Macrophages play important roles in host anti-infection and immune regulation as well. Previous studies have shown that COS is recognized and regulated by macrophages to perform biological functions (12, 40). In this study, we selected COSs with different MWs to stimulate macrophages from blunt snout bream head kidney. Then we studied the stimulation effects at the transcription level by high-throughput sequencing and bioinformatics analysis. We hope to provide theoretical support for the application of COS in the prevention and treatment of bony fish diseases.

In our study, we first demonstrated that GlcNAc could not activate macrophages (10 DEGs) in resting state, and three different MW COSs (COS3, COS6, COS13-19) had a large difference in the number of DEGs after stimulation of macrophages for 4 h. The proportion of up- and downregulated unique genes in the three groups was relatively high. In addition, the genes of different treatment groups had their own unique expression patterns. These results suggest that COSs with different MWs have different biological functions in activating macrophages, which is similar to the study in mammals (4, 41, 42). To investigate the functional similarities and differences of these EDGs in the three groups, biological process GO terms and GSEA KEGG pathway enrichment analysis were used. The results showed that the GO terms' defensive response, inflammatory response, response to bacterium, response to biotic stimulus, response to external biotic stimulus, and response to other organisms were co-activated in the COS3, COS6, and COS13-19 groups. However, the results of GSEA KEGG pathway enrichment showed that the only pathway of co-activation was DNA replication. In addition, COS3 and COS13-19 could activate some infectious disease pathways, COS6 pathways mainly related to DNA replication, and mismatch repair. These results indicated that although COSs have different functions in activating macrophages, they all can activate macrophages to produce inflammation and stress responses in the early stage of stimulation. Notably, DNA replication was the most markedly enriched pathway in the three groups, this may imply that there are other potential biological functions of COSs in stimulating macrophages of blunt snout bream head kidney.

Gene co-expression network analysis (WGCNA) is more likely to identify modules containing co-regulatory genes whose encoded proteins are directly involved in structural units (31). In order to continue to study the functional characteristics of COSs, WGCNA analysis was used to determine the most relevant positive-related modules for COS3, COS6, and COS13-19, and respectively, the magenta, midnightblue and blue modules were determined. The core genes of the magenta module are related to oxygen concentration (15), and the GO term enrichment results focus on protein modification, metabolism, nucleic acid transport, and oxygen reduction processes. These results suggest that COS3 has a weak ability to activate the immune system of resting macrophages at the early stage, and is mainly in the oxidative emergency stage to counteract the invasion of foreign substances (13). Innate immune-related GO terms appear in the midnightblue module, whose core genes are mainly related to innate immunity and cell proliferation (43). Interestingly, the GO terms enriched in the blue module are mainly related to cell growth and death, and the core genes are related to innate immunity and antitumor activity (43, 44). In order to obtain more accurate pathway and protein families and domain enrichment in the blue module, the STRING database was applied to screen genes with high connectivity in the PPI network. KEGG pathway enrichment results showed that the top 10 pathways were mainly related to infectious diseases, the immune system, cell growth and death, signal transduction and metabolism. Then the Pfam database was used to enrich the domains of screened genes, and three protein families were enriched, namely caspase recruitment domain, bZIP (basic leucine zipper) transcription factor, and PH domain. The caspase protein family is associated with cell death, and caspase 1 and caspase 3 are the key genes of pyroptosis and apoptosis, respectively (45, 46). The bZIP transcription factors are involved in many essential cellular processes, and many are associated with cancer. For example, the activator protein 1 (AP-1) family, which includes the well-known transcription factors c-Fos and c-Jun (enriched in the blue module), is responsible for regulation of cell proliferation and apoptosis (47). Apoptosis and the p53 signaling pathway were enriched in the KEGG pathway, which may imply that the p53 pathway is involved in the apoptosis response of macrophages stimulated by COS13-19. Notably, the cell cycle pathway was also enriched and, combined with the enrichment of the DNA replication pathway of GSEA KEGG analysis, we conjectured that DNA replication activates the P53 pathway, causing macrophage apoptosis (38, 48).

Through bioinformatics analysis, we identified traits associated with three different MW COS-stimulated macrophages in the early stage. The MAPK and NF-κB signaling pathways are the two most widely studied pathways related to inflammation in the application of COSs in mammals (4). In our results, the MAPK and NF-κB upstream signaling pathways were enriched in the COS13-19 group. To verify whether these traits were specific in the COS13-19 group, we extended the stimulation time to 16 hours. MAPK is composed of three downstream mediators including C–Jun N–terminal kinase (JNK), extracellular signal-regulated kinase (ERK1/2) and P38 MAPK. These three mediators promote nuclear translocation of AP-1, which induces the transcription of pro-inflammatory genes (4). Our results showed that p38β was first activated at 4 h, then the other molecules of the MAPK pathway were activated, and the activation intensity increased with the increase of stimulation time. NF-κB2 was first upregulated in the NF-κB pathway, then the downstream TNFα gene was upregulated, and the upregulation was most pronounced in the COS13-19 group. These results indicated that the activation degree of macrophages was positively correlated with the MW and duration of stimulation of COS. This trend was also observed in the subsequent detection of cell apoptosis and the identification of apoptotic pathway genes. ATM and ATR are members of the inositol triphosphate kinase family, which sense different forms of DNA damage (49). ATM plays a “checkpoint” role in double-stranded DNA damage. ATR is responsible for sensing and transmitting other forms of DNA damage, including replication fork damage, DNA cross-linking, and so on. These two genes are relatively independent and cross-talk, co-activating downstream p53 pathway proteins. In the DNA replication pathway, MCM family proteins induce cell apoptosis in terminally differentiated cells (Figure S1) (50). Activated macrophages are terminal differentiated cells (51, 52). In our results, ATR and ATM are activated, which may be caused by abnormal replication of DNA, thus activating p53 and downstream proteins, causing cell apoptosis. In addition, the MAPK pathway is important to involve in the initiation of apoptosis. It can be activated by extracellular stimulation to exert biological effects, regulate different cellular functions, and mediate mainly physiological functions such as differentiation, proliferation, and apoptosis. On the one hand, JNK/p38 MAPK signaling activation can increase P53 phosphorylation to regulate the P53 pathway. On the other hand, high expression of AP-1 mainly through bZIP binds DNA to promote the expression of pro-apoptotic genes such as P53, Bax, Fasl, TNF, and so on (22, 53, 54). Apoptosis-related genes in the P53 pathway were significantly upregulated at the late stage of stimulation in three groups (16 h).

In conclusion, we first used high-throughput sequencing and bioinformatics analysis methods to systematically analyze the biological functions of COSs with different MWs on macrophages in teleost. Our results showed that COSs could activate the stress response of macrophages at the early stage, and gradually activate the innate immune response with the increase of stimulation time to resist the invasion of foreign substances, and the activation degree of macrophages was positively correlated with the MW and stimulation time. In addition, we also found that COSs could induce apoptosis of macrophages via the P53 pathway. Firstly, activated macrophages are terminally differentiated cells, and abnormal DNA replication activates ATR and ATM genes to regulate the P53 signaling pathway. Secondly, sustained activation of MAPK pathway genes upregulates P53 expression and phosphorylation, eventually leading to macrophage apoptosis. Inevitably, many immunopotentiators used in fish experiments induce beneficial effects, such as disease protection due to increased cellular and humoral responses. However, attention must be paid to problems such as tolerance, unwanted side effects (e.g., immunosuppression of excessive doses of immunopotentiators) or undesirable effects caused by prolonged use of such compounds.

## Data Availability

Publicly available datasets were analyzed in this study. This data can be found here: https://academic.oup.com/gigascience/article/6/7/gix039/3847731.

## Ethics Statement

The study was approved by the Institutional Animal Care and Use Ethics Committee of Huazhong Agricultural University.

## Author Contributions

XL and CW conceived and designed the experiments. CW and YD performed the experiments and analyzed the data. CW, XL, JS, and GY wrote the manuscript. All authors reviewed the manuscript.

### Conflict of Interest Statement

The authors declare that the research was conducted in the absence of any commercial or financial relationships that could be construed as a potential conflict of interest.

## Supplementary Material

Figure S1 MA plots provided a global view of all differential genes of GlcNAc **(A)**, COS3 **(B)**, COS6 **(C)**, and COS13-19 **(D)** groups, respectively. The mean of normalized counts on the x-axis, the log2 fold change on the y-axis. Red dots represent differentially expressed genes (*P* < 0.001) (29).

Figure S2 Gene set enrichment analysis (GSEA) identified DNA replication pathway was activated in the COS3 **(A)**, COS6 **(B)**, and COS13-19 **(C)** groups. In this plots, genes are ranked by signal/noise ratio according to their differential expression between COS stimulation and control. Genes in the gene set are marked with vertical bars. The normalized enrichment score (NES), nominal *P*-value and FDR are shown in these plots. The heatmap on the right reflects the expression of DNA replication-related genes in different stimulation groups.

Figure S3 The bright field of immunofluorescence staining. Annexin V is the green fluorescent marker, labeling cells in early apoptosis. PI (Propidium Iodide) is the red fluorescent marker, labeling cells in late apoptosis. Bars: 25 μm.
