# Supplementary figures and images for: Immunomodulatory Effects and Induction of Apoptosis by Different Molecular Weight Chitosan Oligosaccharides in Head Kidney Macrophages From Blunt Snout Bream (Megalobrama amblycephala)
Source: Front Immunol. 2019 May 15;10:869. doi: 10.3389/fimmu.2019.00869 (PMC6530513; doi:10.3389/fimmu.2019.00869)

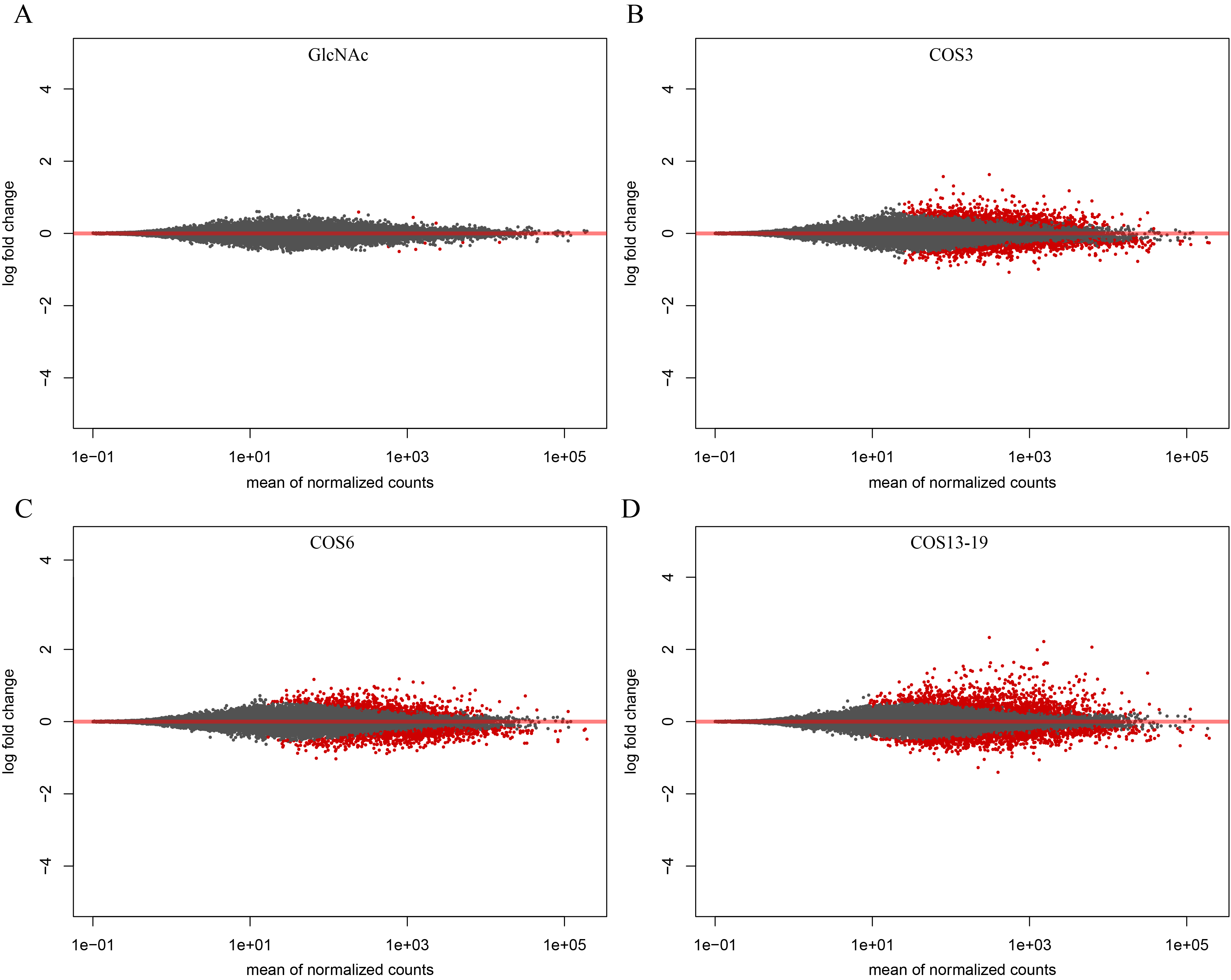

Supplement: Figure S1 — MA plots provided a global view of all differential genes of GlcNAc (A), COS3 (B), COS6 (C), and COS13-19 (D) groups, respectively. The mean of normalized counts on the x-axis, the log2 fold change on the y-axis. Red dots represent differentially expressed genes (P < 0.001) (29). [file Image_1.tif]

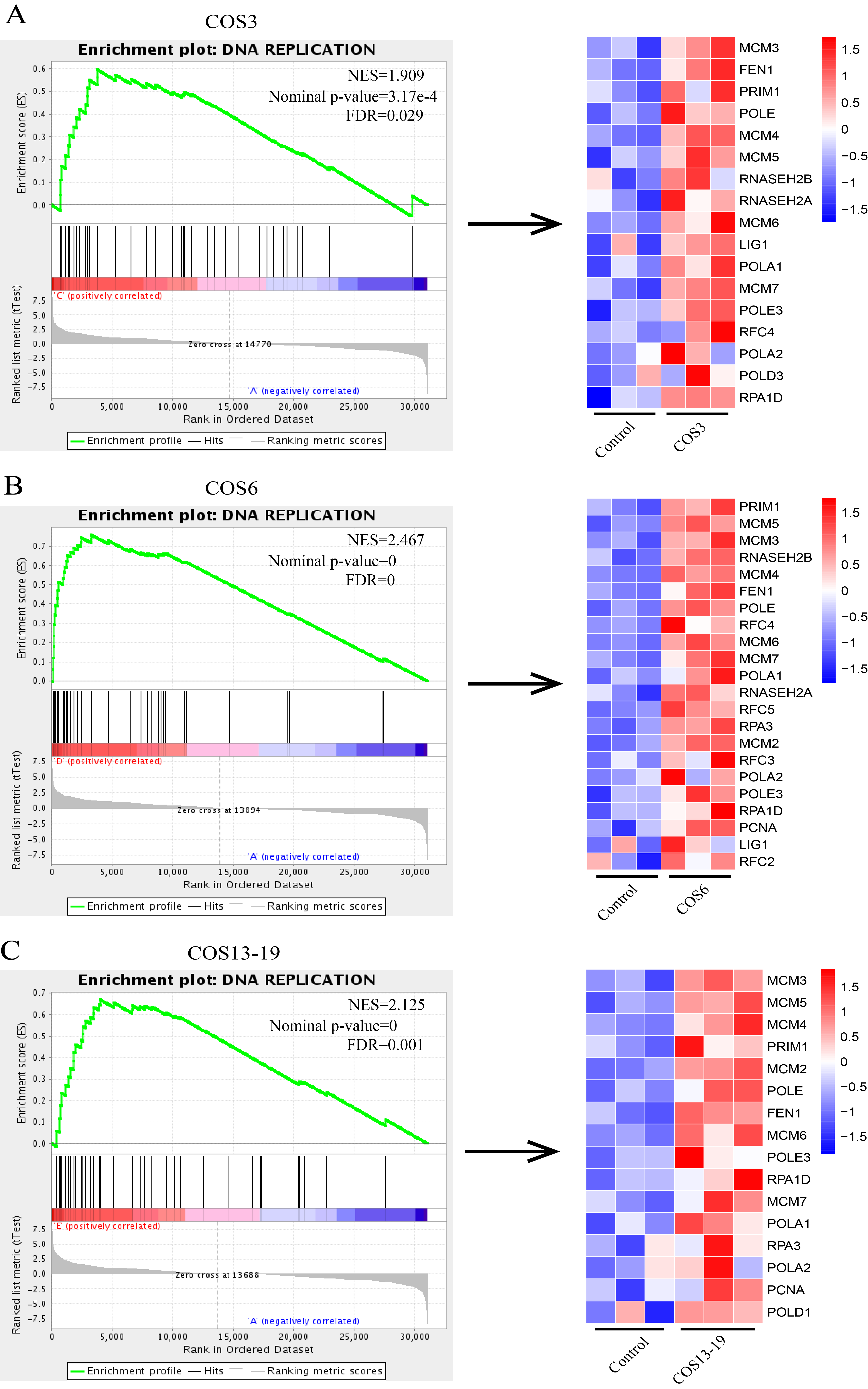

Supplement: Figure S2 — Gene set enrichment analysis (GSEA) identified DNA replication pathway was activated in the COS3 (A), COS6 (B), and COS13-19 (C) groups. In this plots, genes are ranked by signal/noise ratio according to their differential expression between COS stimulation and control. Genes in the gene set are marked with vertical bars. The normalized enrichment score (NES), nominal P-value and FDR are shown in these plots. The heatmap on the right reflects the expression of DNA replication-related genes in different stimulation groups. [file Image_2.tif]

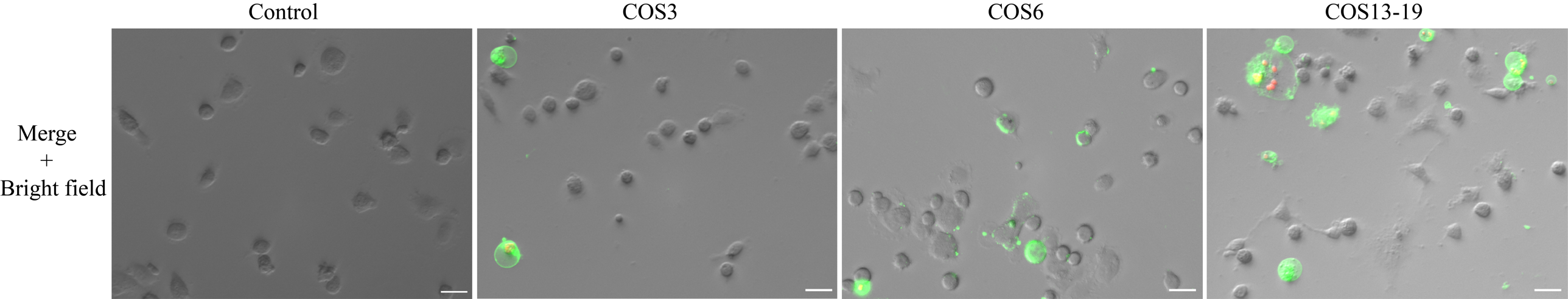

Supplement: Figure S3 — The bright field of immunofluorescence staining. Annexin V is the green fluorescent marker, labeling cells in early apoptosis. PI (Propidium Iodide) is the red fluorescent marker, labeling cells in late apoptosis. Bars: 25 μm. [file Image_3.tif]
